# Psychiatric disorders following the clustering of family disadvantages in previous generations: a multigenerational cohort study

**DOI:** 10.1007/s00127-025-02918-z

**Published:** 2025-04-29

**Authors:** Baojing Li, Can Liu, Ylva B. Almquist, Lisa Berg

**Affiliations:** Department of Public Health Sciences, Centre for Health Equity Studies (CHESS), Stockholm University, Stockholm, SE-106 91 Sweden

**Keywords:** Socioeconomic factors, Psychosocial factors, Mental health, Multigenerational transmission, Sweden

## Abstract

**Purpose:**

There is a lack of multigenerational research on the extent to which mental health is informed by transmission of multiple disadvantages across previous generations. This study aims to investigate how family socioeconomic and psychosocial disadvantages cluster and transition over grandparental and parental generations, and how this might be associated with grandchild psychiatric disorders.

**Methods:**

We utilized a cohort study with data following three generations from the Stockholm Birth Cohort Multigenerational Study, including 11,299 individuals born in 1953 (parental generation), their 22,598 parents (grandparental generation), and 24,707 adult children (grandchild generation). Family disadvantages as exposures were measured across two periods– grandparental adulthood (parental childhood) and parental adulthood (grandchild childhood), and included socioeconomic (i.e., low income, non-employment, overcrowding, and single parenthood) and psychosocial aspects (i.e., single parenthood, teenage motherhood, psychiatric disorders, and criminality of father). Psychiatric disorders in the adult grandchildren as outcome were defined by hospitalizations with a main or contributing diagnosis reflecting mental and behavioral disorders from age 18 until 2019.

**Results:**

Multiple disadvantages *within* the grandparental and parental generations, respectively, predicted higher probabilities of grandchild psychiatric disorders. Multigenerational transmission is evident in that grandchildren with combinations of grandparental socioeconomic disadvantages and parental psychosocial disadvantages had comparably high probabilities of psychiatric disorders. Importantly, improved socioeconomic and psychosocial circumstances *across* previous generations predicted comparably low probabilities of grandchild psychiatric disorders.

**Conclusion:**

Mental health of future generations is informed by the transmission of multiple disadvantages across previous generations, and the transition from grandparental socioeconomic disadvantages into parental psychosocial disadvantages is particularly important.

**Supplementary Information:**

The online version contains supplementary material available at 10.1007/s00127-025-02918-z.

## Introduction

Mental health problems are important causes of disease burden [1, 2] and have been increasing in many high-income countries, including Sweden [3, 4]. Previous research suggests that early-life socioeconomic and psychosocial disadvantages can increase the likelihood of later mental health problems among offspring [5]. Higher risks of mental health problems have been found in socioeconomically disadvantaged families, characterized by low parental educational attainment [6], low parental income [7], and low-quality housing conditions [8]. Moreover, family psychosocial disadvantages, such as young motherhood [9], single parenthood [10], and parental imprisonment [11], may also play a role in elevating the risks of offspring mental health problems. These family socioeconomic and psychosocial processes could be broadly explained by the Family Stress Model (FSM), which posits that economic hardship gives rise to greater emotional distress and family conflict among parents, ultimately contributing to offspring mental health problems [12]. Notwithstanding, without denying that studies focusing on relations between different variables of disadvantage and mental health can be useful in studying complex associations, it is clear that this approach neglects the crucial importance of a holistic and dynamic view of the individual as an integrated totality who might have experienced different forms of family disadvantages over time [13]. Families with certain concentration or combinations of socioeconomic and psychosocial disadvantages may struggle to build the human capital, especially in terms of mental health [14], needed for the offspring to move beyond their level of disadvantage, thereby leading to persistent or widening inequalities [15]. A person-centered approach, whereby conceptualizing the population as a mixture of subgroups of individuals sharing similar profiles of family disadvantages, could capture these clustering patterns [16]. This approach adds value for understanding the complexity in how and for whom certain disadvantage clusters, as well as how and for whom these co-occurring disadvantages affect offspring mental health.

While research into the clustering of family disadvantages and the role it plays for offspring mental health problems can provide important insights, extending parent-child relationships into a multigenerational framework might further our understanding of how disadvantages are transmitted across generations to give rise to inequalities in offspring mental health. Based on cumulative (dis)advantage theory [17, 18], it might be hypothesized that multiple disadvantages among grandparents persist or even become magnified in the parental generation, thus imposing further disadvantages on grandchildren’s mental health. Nevertheless, it is possible that specific transition patterns across the grandparental and parental generations do not align with this general expectation. In some cases, resilience processes may emerge, leading to intergenerational upward mobility where the parental generation attains a higher status than the grandparental generation [19]. Although studies have examined intergenerational patterns of disadvantage transmission using a person-centered approach [20, 21], no study has specifically focused on the role of such intergenerational patterns in third-generation mental health. Finally, there may also be gender differences in mental health problems among the grandchildren due to varying exposures to disadvantages and underlying vulnerabilities, as well as differential access to resources and opportunities, and differences in the ability to respond to disadvantages [22].

Against this background, the current study will adopt a person-centered approach to the exploration of how family socioeconomic and psychosocial disadvantages cluster and transition over grandparental and parental generations, and how this might be associated with psychiatric disorders among adult male and female grandchildren. We address the following research questions:

1. What kind of clusters of family disadvantages within the grandparental and parental generations can be identified, and how can transition patterns of these clusters across the grandparental and parental generations be characterized?
2. How are the clusters of family disadvantages within the grandparental and parental generations, respectively, associated with psychiatric disorders among grandchildren, and do these associations differ by grandchild gender?
3. In what way do the probabilities of psychiatric disorders among grandchildren vary depending on the transition patterns of the clusters of family disadvantages across the grandparental and parental generations, and are there any differences by male and female grandchildren?

## Materials and methods

### Study population

**Fig. 1:**
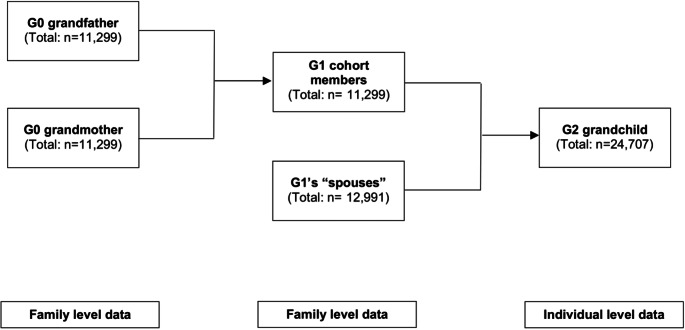
Multigenerational data structure for the study population. G1’s “spouses” specifically refers to G2 grandchild’ another biological parent (either mother or father)

Data were extracted from the Stockholm Birth Cohort Multigenerational Study (SBC Multigen), which is based on two de-identified data materials: RELINK53 and the Stockholm Metropolitan Study (SMS) [23]. SBC Multigen is defined as all individuals born in 1953 and living in the greater Stockholm metropolitan area in 1963 from the SMS, who could be probability matched to the follow-up nationwide register data from RELINK53 (*n* = 14,608). The Regional Ethical Review Board in Stockholm approved the creation of RELINK53 and the SBC Multigen (no. 2017/34–31/5; 2017/684–32). This study was approved by the Swedish Ethical Review Authority (no. 2021-00090). We utilized a cohort study design with individual- and family-level data for three generations, centered around the 11,299 individuals born in 1953 (G1, parental generation), their 22,598 parents (G0, grandparental generation; birth years: 1877–1941), and 24,707 children (G2, grandchild generation; birth years: 1968–2001). The analytic sample for the current study was restricted to G2s who had reached 18 years of age by 2019 and for whom both biological parents had been identified. Details on the multigenerational data structure are shown in Fig. 1.

### Measures

Information on psychiatric disorders was obtained from the inpatient register of the National Patient Register. G2s were defined as having the outcome of interest– psychiatric disorders– if they had records of inpatient care with a main or contributing diagnosis reflecting mental and behavioral disorders from age 18 up until 2019 when the oldest G2 turned age 51 (Chapter F in the International Classification of Diseases, 10th revision [ICD 10], and the corresponding chapters in the 9th revision [ICD 9]), excluding those disorders with an early onset and typically with a strong biological basis (ICD 10: F70-F98 and corresponding ICD 9 codes) that did not co-occur with other diagnoses of mental and behavioral disorders (*n* = 1,821). Family disadvantages as exposures were measured across two time periods– G0’s adulthood (G1’s childhood) and G1’s adulthood (G2’s childhood). Details on the measurements of family disadvantages, including socioeconomic (i.e., low income, non-employment, overcrowding, and single parenthood) and psychosocial aspects (i.e., single parenthood, teenage motherhood, psychiatric disorders, and criminality of father), as well as descriptive statistics and missing data are presented in Table 1.

**Table 1:** Measurements, descriptive statistics and missing data for the study variables among the grandparental (G0) and parental (G1) generations

Family disadvantages (Variable type)	G0				G1			
	Definitions	Data sources	Numbers (proportions)	Missing data (proportions)	Definitions	Data sources	Numbers (proportions)	Missing data (proportions)
Low income (binary variable, at or below (including no income) versus above the lowest 25th percentile)	Highest total net income from either G1’s father or mother in 1963.	Register of population and income (part of the SMS)	6,400 (25.0%)	0 (0.0%)	Highest disposable income from either G2’s father or mother in 1990.	Longitudinal Integration Database for Health Insurance and Labour Market Studies (LISA, part of RELINK53)	5,261 (20.7%)	259 (1.0%)
Non-employment (binary variable)	Head of the household being economically inactive, and unemployed (housewives, studies, military service, illness and disablement, others) in 1960.	The 1960 Census (part of the SMS)	1,982 (8.3%)	1,639 (6.4%)	Either G2’s father or mother (or both of them) being unemployed (student, military service, or other) in 1990.	The 1990 Census (part of RELINK53)	2,280 (9.8%)	2,314 (9.0%)
Overcrowding (binary variable)	More than two persons per room (kitchen not included) in 1960.	The 1960 Census (part of the SMS)	4,296 (18.0%)	1,804 (7.0%)	More than two persons per room (kitchen and one room not included) in 1990.	The 1990 Census (part of RELINK53)	1,368 (6.2%)	3,396 (13.3%)
Single parenthood (binary variable)	Either “G1’s mother living alone” or “G1’s father living alone” (or both of them) in 1964.	Register of population and income (part of the SMS)	2,311 (9.0%)	9 (0.0%)	Either “single father” or “single mother” of G2 (or both of them) in 1990.	LISA (part of RELINK53)	4,627 (18.5%)	593 (2.3%)
Teenage motherhood (binary variable)	G1’s mother who gave birth at or below age 19, calculating mother’s age at birth for the index G1 child.	Total Population Register (part of RELINK53)	1,259 (5.0%)	178 (0.7%)	G2’s mother who gave birth at or below age 19, calculating mother’s age at birth for the index G2 child.	Total Population Register (part of RELINK53)	1,035 (4.3%)	1,453 (5.7%)
Parental psychiatric disorders (binary variable)	Either G1’s father or mother (or both of them) having pre-categorized information about alcohol abuse, psychiatric problems, depression, as well as psychiatric treatment.	Locally kept Social Registers (part of the SMS)	2,204 (8.6%)	43 (0.2%)	Either G2’s father or mother (or both of them) having records of inpatient care with a main or contributing diagnosis reflecting mental and behavioral disorders from the ICD 10 (F00-F69; F99), and the corresponding ICD 9.	National Patient Register (part of RELINK53)	2,557 (10.0%)	0 (0.0%)
Criminality of father (binary variable)	Conditional sentence (including fine/probation), unconditional sentence (imprisonment), drink-driving/dangerous driving and exemption from punishment due to institutional psychiatric care or alcohol treatment.	National Crime Register (part of the SMS)	2,009 (7.8%)	0 (0.0%)	Prosecution decisions from a court judgment or prosecution decisions made outside the court, such as approved penal orders or waived prosecutions (fines or penalty notices issued by the police or customs are not included).	National Crime Register (part of RELINK53)	5,300 (20.7%)	0 (0.0%)

### Statistical analyses

As the first step, latent class analysis (LCA) was used to determine latent classes among G0s and G1s, separately. The units of analysis were G2s. LCA is a statistical procedure used to identify meaningful subgroups based on combinations of response patterns across a set of observed variables [24, 25]. All binary indicators of socioeconomic and psychosocial disadvantages were included in the LCA models. The number of latent classes was progressively increased by one class in step-wise models; from one to six latent classes. We considered multiple fit statistics, including Akaike information criterion (AIC), Bayesian information criterion (BIC), Sample adjusted Bayesian information criterion (SABIC), Entropy, p-value of the Voung-Lo-Mendel-Rubin likelihood ratio test (VLMR-LRT), minimum number of sample members in each class (and proportions), and theoretical interpretability for choosing the number of optimal classes [26]. Among these, BIC is by many considered to be the most reliable fit statistic, Entropy serves as a classification diagnostics, and p-value of the VLMR-LRT indicates if one model is statistically better than another [26]. Furthermore, latent transition analysis (LTA) was conducted [27], based on the latent class indicators among G0s and G1s to estimate the transition probabilities of family disadvantages over grandparental and parental generations. We produced a river plot to visualize the transition process.

In the second step, each G2 was assigned with the most appropriate G0 and G1 latent classes using proportional Bolck–Croon–Hagenaars (BCH) adjustments to predict class membership. The BCH adjustment method accounts for potential classification errors; specifically, the association between the latent classes and the distal outcome is computed by using the joint probability distribution of the assigned class membership and the distal outcome, weighted by the inverse of the matrix of the misclassification probabilities [27–29]. Thereafter, based on logistic regression analysis, we estimated the probabilities of grandchild psychiatric disorders within grandparental and parental latent classes, respectively, and the probabilities of grandchild psychiatric disorders for across each of the grandparental and parental latent classes. We further stratified these models by grandchild gender. As for sensitivity analyses, we re-ran the analyses (1) for the same sample but G2s were followed up to age 30, and (2) adjusting for the clustering/non-independence of observations within families.

The full information maximum likelihood approach has been adopted as an efficient way of handling missing data for modelling algorithms such as LCA and LTA [30]. Data were managed using Stata SE 17, all analyses were performed in Mplus Version 8, and artwork was created with Excel and R software version 4.3.0.

## Results

**Fig. 2:**
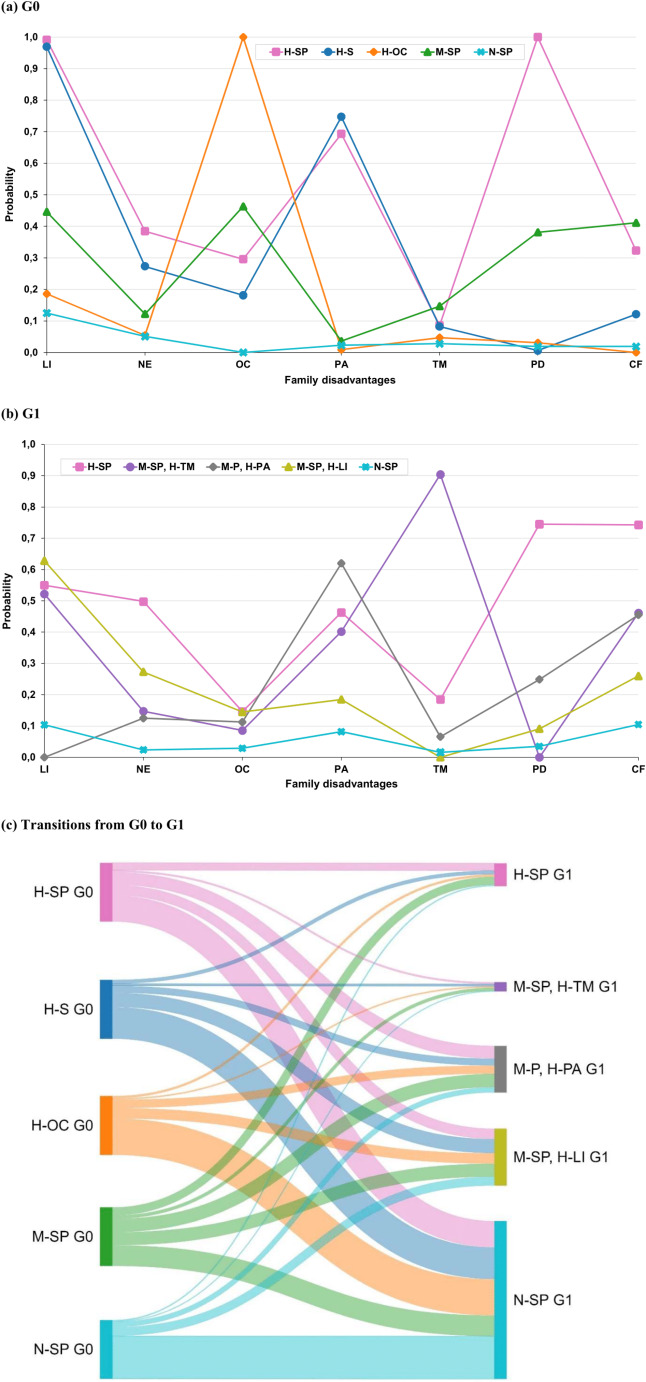
Estimated probabilities of family disadvantages across grandparental (**a**, G0) and parental (**b**, G1) latent classes, and transition probabilities from G0 to G1 (**c**) latent classes of family disadvantages. Results from latent class analysis and latent transition analysis. LI: Low income; NE: Non-employment, OC: Overcrowding; PA: Single parenthood; TM: Teenage motherhood; PD: Psychiatric disorders; CF: Criminality of father. For G0, H-SP: High-level socioeconomic and psychosocial disadvantages; H-S: High-level socioeconomic disadvantages; H-OC: High-level overcrowding; M-SP: Medium-level socioeconomic and psychosocial disadvantages; N-SP: No socioeconomic and psychosocial disadvantage; For G1, H-SP: High-level socioeconomic and psychosocial disadvantages; M-SP, H-TM: Medium-level socioeconomic and psychosocial disadvantages, with high-level teenage motherhood; M-P, H-PA: Medium-level psychosocial disadvantages, with high-level single parenthood; M-SP, H-LI: Medium-level socioeconomic and psychosocial disadvantages, with high-level low income; N-SP: No socioeconomic and psychosocial disadvantage

The labelling of different G0 and G1 latent classes is based on an assessment of estimated probabilities across the different indicators of socioeconomic and psychosocial disadvantages. The five-class solution was selected as the best model for G0 latent classes, with the highest entropy of 0.817 and a relatively low AIC, BIC, and SABIC (Online Resource 1). Fig. 2a presents the estimated probabilities of family disadvantages across G0 latent classes. The first latent class was labeled as “High-level socioeconomic and psychosocial disadvantages” (H-SP, 2.5%). “High-level socioeconomic disadvantages” (H-S, 7.0%) was the label for the second latent class. The third latent class was characterized by “High-level overcrowding” (H-OC, 10.7%). The fourth latent class had “Medium-level socioeconomic and psychosocial disadvantages” (M-SP, 11.9%). The fifth latent class represented “No socioeconomic and psychosocial disadvantage” (N-SP, 68.0%).

For G1 latent classes, the five-class solution, which had a reasonable model fit with the lowest BIC and a sufficient entropy of 0.630, was also considered as the best model (Online Resource 1). Fig. 2b presents the estimated probabilities of family disadvantages across G1 latent classes. The first latent class indicated “High-level socioeconomic and psychosocial disadvantages” (H-SP, 4.4%). The second latent class was labelled as “Medium-level socioeconomic and psychosocial disadvantages, with high-level teenage motherhood” (M-SP, H-TM, 1.9%). The third latent class represented “Medium-level psychosocial disadvantages, with high-level single parenthood” (M-P, H-PA, 11.7%). The fourth latent class was characterized by “Medium-level socioeconomic and psychosocial disadvantages, with high-level low income” (M-SP, H-LI, 16.8%). The fifth latent class had “No socioeconomic and psychosocial disadvantage” (N-SP, 65.2%).

Fig. 2c Shows that each of the G0 classes was most likely to transition to the G1 class having no socioeconomic and psychosocial disadvantage (N-SP). Transition probability estimates are shown in Online Resource 2.

Table 2 shows the probabilities of psychiatric disorders among G2s by G0 and G1 latent classes, respectively. G2s who originated from the G0 class with medium-level (M-SP) (0.118, 95% CI − 0.100 to 0.136) and G1 class with high-level (H-SP) (0.180, 95% CI − 0.143 to 0.217) socioeconomic and psychosocial disadvantages had the highest probabilities of psychiatric disorders. The second highest probabilities of psychiatric disorders were found for G2s in the G0 class with high-level socioeconomic disadvantages (H-S) (0.097, 95% CI − 0.080 to 0.113) and G1 class in medium-level psychosocial disadvantages, with high-level single parenthood (M-P, H-PA) (0.139, 95% CI − 0.115 to 0.163). Overall, the patterns were similar for G2 males and females. In the sensitivity analysis using the same sample with G2s followed up until age 30, results were similar to those from the main analysis, with a few exceptions: (1) G2 females in the G0 classes with M-SP and H-S still had the top two highest probabilities of psychiatric disorders, but the ranking of these two classes was reversed; and (2) G2 females in the G1 class with M-P, H-PA turned to have the highest probabilities of psychiatric disorders (Online Resource 3).

**Table 2:** Probabilities of psychiatric disorders among grandchildren (G2) by grandparental (G0) and parental (G1) latent classes of family disadvantages. Results from logistic regression analysis

Family classes		Psychiatric disorders probability		
		Total	Male	Female
		Probability (95% CI)	Probability (95% CI)	Probability (95% CI)
	H-SP	0.078 (0.051–0.104)	0.086 (0.046–0.125)	0.070 (0.034–0.105)
	H-S	0.097 (0.080–0.113)	0.087 (0.065–0.109)	0.107 (0.082–0.132)
G0	H-OC	0.078 (0.066–0.091)	0.081 (0.064–0.099)	0.075 (0.057–0.093)
	M-SP	0.118 (0.100-0.136)	0.126 (0.100-0.151)	0.110 (0.084–0.135)
	N-SP	0.059 (0.055–0.063)	0.055 (0.050–0.060)	0.063 (0.057–0.069)
	H-SP	0.180 (0.143–0.217)	0.210 (0.156–0.263)	0.146 (0.095–0.196)
	M-SP, H-TM	0.127 (0.079–0.174)	0.141 (0.069–0.213)	0.113 (0.050–0.176)
G1	M-P, H-PA	0.139 (0.115–0.163)	0.145 (0.110–0.180)	0.132 (0.099–0.166)
	M-SP, H-LI	0.069 (0.053–0.086)	0.062 (0.040–0.084)	0.078 (0.053–0.103)
	N-SP	0.050 (0.045–0.056)	0.046 (0.038–0.053)	0.055 (0.048–0.063)

CI, confidence interval

For G0

H-SP: High-level socioeconomic and psychosocial disadvantages;

H-S: High-level socioeconomic disadvantages;

H-OC: High-level overcrowding;

M-SP: Medium-level socioeconomic and psychosocial disadvantages;

N-SP: No socioeconomic and psychosocial disadvantage;

For G1

H-SP: High-level socioeconomic and psychosocial disadvantages;

M-SP, H-TM: Medium-level socioeconomic and psychosocial disadvantages, with high-level teenage motherhood;

M-P, H-PA: Medium-level psychosocial disadvantages, with high-level single parenthood;

M-SP, H-LI: Medium-level socioeconomic and psychosocial disadvantages, with high-level low income;

N-SP: No socioeconomic and psychosocial disadvantage

**Fig. 3:**
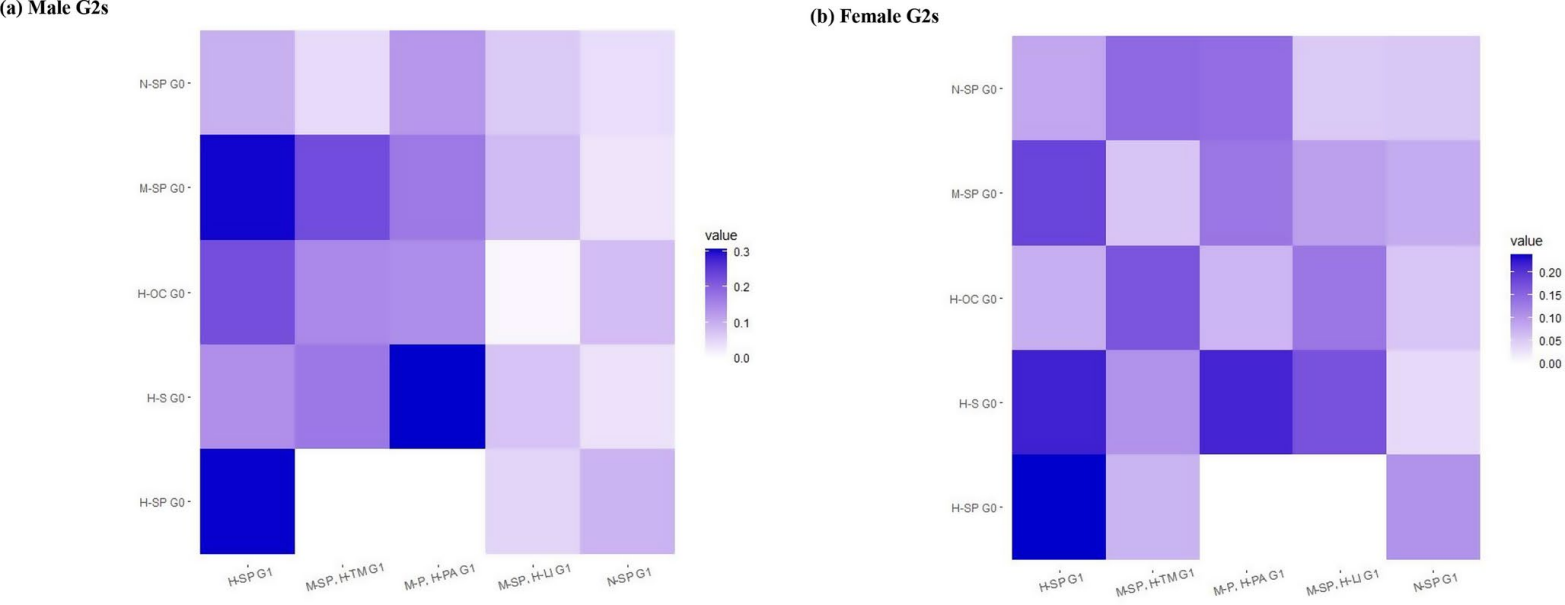
Probabilities of psychiatric disorders among male (a) and female (b) grandchildren (G2) based on the transitions from grandparental (G0) to parental (G1) latent classes of family disadvantages. Results from logistic regression analysis. For G0, H-SP: High-level socioeconomic and psychosocial disadvantages; H-S: High-level socioeconomic disadvantages; H-OC: High-level overcrowding; M-SP: Medium-level socioeconomic and psychosocial disadvantages; N-SP: No socioeconomic and psychosocial disadvantage; For G1, H-SP: High-level socioeconomic and psychosocial disadvantages; M-SP, H-TM: Medium-level socioeconomic and psychosocial disadvantages, with high-level teenage motherhood; M-P, H-PA: Medium-level psychosocial disadvantages, with high-level single parenthood; M-SP, H-LI: Medium-level socioeconomic and psychosocial disadvantages, with high-level low income; N-SP: No socioeconomic and psychosocial disadvantage

The transition pattern– consistently in high-level socioeconomic and psychosocial disadvantages (H-SP) across G0s and G1s– predicted the highest probabilities of psychiatric disorders among female G2s (0.239, 95% CI − -0.043 to 0.521), and the second highest probabilities of psychiatric disorders among male G2s (0.305, 95% CI − 0.058 to 0.552). While the transition pattern– high-level socioeconomic disadvantages (H-S) among G0s and medium-level psychosocial disadvantages, with high-level single parenthood (M-P, H-PA) among G1s– predicted the highest probabilities of psychiatric disorders among male G2s (0.306, 95% CI − 0.097 to 0.514), and the third highest probabilities of psychiatric disorders among female G2s (0.215, 95% CI − 0.022 to 0.409) (Fig. 3a and b; see point estimates and 95% confidence intervals in Online Resource 5). In general, patterns characterized by transitions to G1 class with no disadvantage (N-SP) from all G0 classes (with the exception of G0 class with H-SP) predicted comparably low probabilities of psychiatric disorders among G2s (Fig. 3a and b; see point estimates and 95% confidence intervals in Online Resource 5). In the sensitivity analysis using the same sample with G2s followed up until age 30, the transition pattern– H-S among G0s and M-P, H-PA among G1s– emerged as the predictor of the highest probabilities of psychiatric disorders among both male and female G2s. Meanwhile, the transition pattern– H-SP across G0s and G1s– was no longer the leading driver of probabilities of psychiatric disorders in either G2 gender (Online Resource 6).

## Discussion

We identified five latent classes describing the disadvantageous patterns in the grandparental and parental generations, respectively. Multiple medium- to high-level disadvantages *within* grandparental and parental generations, respectively, predicted higher probabilities of grandchild psychiatric disorders. Multigenerational transmission is evident in that grandchildren with combinations of high-level grandparental socioeconomic disadvantages and parental psychosocial disadvantages (especially single parenthood) had comparably high probabilities of psychiatric disorders. Importantly, improved socioeconomic and psychosocial circumstances *across* previous generations predicted comparably low probabilities of grandchild psychiatric disorders. There was no apparent difference by grandchild gender.

We identified five distinct family disadvantage patterns that parsimoniously captured the variability and intersection of multiple risk factors in the grandparental and parental generations, respectively. From grandparental to parental generations, socioeconomic factors declined in relevance, while psychosocial factors, especially those reflecting family structure such as single parenthood and teenage motherhood, became more relevant in distinguishing different groups. This might reflect the changing structure and norms of the Swedish society. Sweden experienced a great decline in inequality in terms of distribution of income and opportunities from the 1870s until the 1970s, which was driven by the upgrading of the jobs structure, the destruction of capital among the wealthier in the Depression and the Second World War, and the expanding welfare state [31, 32]. Meanwhile, the Swedish society has become more individualistic over time from the 1970s onwards, and there has been substantial progress in achieving gender equality, with an increased focus on individual independence, autonomy, and reliance on welfare-state institutions rather than on the family unit for social and economic security [33]. Specifically, reforms of marriage and family law and policy led to blurring of boundaries between married and cohabiting couples and more accepted family complexity in an era of rising divorce, re-partnering, and stepfamilies [34]. Additionally, Sweden’s dual-earner and dual-carer model emerged, including policies such as individualized taxation, ending marriage subsidies for a dependent spouse, publicly supported daycare provision, and gender-neutral parental leave policy [34, 35].

Our findings on intergenerational associations suggest that offspring from families with a concentration of multiple high-level socioeconomic and psychosocial disadvantages tended to have the highest probabilities of psychiatric disorders. This is consistent with previous research based on LCA, showing the highest internalizing and externalizing problems in classes with the most adverse childhood experiences (i.e., similar measures of disadvantages but with additional dimensions such as child maltreatment and domestic violence) [36]. Internalizing and externalizing problems are both strongly linked to psychiatric disorders, with internalizing traits reflecting emotional distress often associated with mood and anxiety disorders, while externalizing traits manifest as behavioral difficulties commonly linked to conduct disorders [37]. The strong presence of these traits in disadvantaged groups highlights the enduring impact of early-life adversity on later mental health. The parental latent class with particularly high-level single parenthood was also found to predict the second highest probabilities of psychiatric disorders among offspring. Prior research [38–40] has reported that the association between single parenthood and offspring outcomes tends to be weak and is often explained by associated social and contextual factors. However, our findings emphasize the psychosocial aspect of single parenthood by showing that the parental latent class with high-level single parenthood, but low-level socioeconomic disadvantages, strongly contributed to offspring psychiatric disorders. One potential explanation could be that psychosocially disadvantaged families, characterized by single parenthood, parental psychiatric disorders, and fathers’ criminality, struggle to provide adequate psychosocial support for their children. Consequently, children may experience poor psychological adjustment, and psychological and social difficulties associated with recurring stressors, which further contribute to the development of psychiatric problems in later life [41]. Specifically, considering the Swedish context, as discussed above, the increasingly individualistic society and policies promoting gender-neutral parental equality have fostered the practice of joint physical custody [42]. Thus, the parental group identified as having high-level single parenthood is likely to be more mixed in terms of different physical custody forms due to the increasingly common joint physical custody compared to the grandparental generation. It is therefore an important direction for future research to disentangle how different physical custody forms among separated parents affect offspring mental health.

This study extends previous knowledge by demonstrating the important role of family disadvantages in the generation preceding one’s immediate family. Among the grandparental latent classes, the high-level socioeconomic disadvantages class (i.e., low income, non-employment, overcrowding, and single parenthood in terms of the socioeconomic aspect) was found to predict high probabilities of grandchild psychiatric disorders. This finding can, to some extent, be interpreted in light of the Family Stress Model (FSM) [43]. FSM explains the impact of financial hardship through the complex nature of family’s experiences with links between economic pressure, parental psychological distress, marital conflict, and disrupted parenting. These disruptions are expected to “spill over” into interactions with children, thereby affecting their mental health outcomes [44]. From a multigenerational perspective, FSM has also been cross-validated in samples of custodial grandparents [45], who have played the role as parents. It nonetheless still remains unclear whether this can be applied to grandparents in general, regardless of whether they are raising their grandchildren.

We identified a key transition pattern across previous generations as the leading predictor of comparably high probabilities of grandchild psychiatric disorders– combinations of grandparental socioeconomic disadvantages and parental psychosocial disadvantages, with high-level single parenthood. This finding suggests that grandparental socioeconomic and parental psychosocial disadvantages jointly contribute to poor mental health outcomes among offspring, and is supported by a multigenerational extension of cumulative (dis)advantage theory. In additional to the broad range of environmental factors we have discussed earlier, genetic factors may be another plausible explanation for our observed inter- and multi-generational results. The associations between family disadvantages experienced by both grandparental and parental generations and grandchild psychiatric disorders could be results of inter- and multi-generational transmission of psychiatric morbidity attributable to shared genetic factors across generations [46]. However, genetic factors obviously cannot fully account for the observed variations of offspring psychiatric morbidity by different socioeconomic and psychosocial situations of previous generations. Another key finding of our study is that patterns characterized by transitions to the parental latent class with no disadvantage, from almost each grandparental latent class, predicted comparably low probabilities of grandchild psychiatric disorders. This finding highlights that if the socioeconomic and psychosocial circumstances of the parental generation are good, grandchildren will have relatively low risks of psychiatric disorders irrespective of the disadvantages from the grandparental generation, or, put differently, that upward social mobility across previous generations (i.e., from grandparents facing disadvantages to parents without such disadvantage) benefits the future generation’s mental health outcomes. On the other hand, grandchildren whose parents are disadvantaged seem to benefit from having better-off grandparents. To sum up, understanding transmission pathways of multiple disadvantages across previous generations is an important part of being able to inform policies and interventions alleviating the burden of family disadvantages for improving the mental health of subsequent generations. Intervening on only one type of disadvantage would likely not suffice, and instead there is a need for comprehensive support systems that address various dimensions of disadvantage simultaneously. Thus, a more effective intervention strategy would involve implementing multiple, integrated, and coordinated approaches that go beyond addressing isolated indicators of disadvantages [15]. Such intervention approaches should particularly focus on enhancing parents’ psychosocial well-being, and meanwhile, support from other adults, such as grandparents, may help mitigate parental disadvantages and contribute to positive offspring mental health outcomes.

### Methodological considerations

Our study has several key strengths. It builds on a multigenerational data material that encompasses local and national survey- and register-based data for a relatively large, community-based sample. This allows us to measure disadvantages across multiple life domains over three subsequent generations in a largely comparable way. This is reflected in the relatively similar proportions of different disadvantage indicators across grandparental and parental generations (Table 1), with the exception of single parenthood and paternal criminality, which align with the historical trends of increasing rates in the Swedish context [34, 47]. While single parenthood became increasingly relevant in distinguishing different groups from grandparental to parental generations, paternal criminality did not seem to play an important role in distinguishing different classes, as it increased across almost all latent classes from grandparental to parental generations rather than being concentrated in one specific class (Fig. 2b), as a result, its effect on the latent class structure, and consequently on the overall results, is likely minimal. Meanwhile, applying a person-centered approach enables us to capture multidimensional disadvantages that cluster within the family context. We also acknowledge important limitations. First, we only captured a selection of disadvantages. For example, because the education-related variable in the 1960 census yielded a large proportion of grandparents (around 70%) to be labelled in the low education group (i.e., neither head of the household nor wife has any upper secondary education), we had to exclude education indicators for both grandparents and parents to ensure the comparability across these two generations. Future research should utilize a wider range of information (e.g., education, attitudes and beliefs, and family relationships), and further examine the role of other possible disadvantages facing families in defining clusters and in shaping subsequent offspring mental health outcomes. Second, regarding our outcome– psychiatric disorders, we only used data from the inpatient register to ensure adequate data coverage for the grandchild population. The number of hospital discharges in the inpatient register has remained stable since 1973, while the outpatient register only commenced in 2001 and the prescribed drug register only established in 2005 (i.e., when the oldest grandchild turned 33 in 2001 and 37 in 2005, and information between age 18 and 33-37 would be missing) [48]. Thus, we were only able to capture the most severe cases, which might lead to underestimation of the prevalence of these mental health conditions in the population. Moreover, we did not have access to data from primary health care, where a large proportion of mental disorders are treated [49]. Third, while many psychiatric disorders are considered to have a hereditary component [50], we have no access to genetic information, and thus it is impossible to rule out biological predisposition of grandchild psychiatric disorders. Fourth, although there might be biases due to clustering/non-independence of observations within families (i.e., grandchildren are clustered within parents), the sensitivity analyses adjusting for such clustering yielded similar estimates, with the largest difference in estimates being 0.001 (Online Resource 4 and 7). Fifth, we only have information on either maternal or paternal grandparents unless both of grandchildren’s parents belong to the original SMS cohort. Maternal grandparents have been found to be more likely to provide support and to be involved in the lives of grandchildren than paternal grandparents [51]. This could potentially introduce bias in the estimation of associations in our study when pooling both lineages. However, given that our study focuses on structural family disadvantages rather than direct caregiving, we believe this limitation is unlikely to substantially affect our main results. Lastly, we have to be aware not to reify the meaning attached to latent classes as a “real” set of categories when interpreting the findings [52]. The final models are only one possible summary of the many ways in which constellations of disadvantages may occur. Furthermore, our findings are specific to the current study sample and the risk factors included in the models, and thus generalizability may be limited, given that different sets of risks, different dichotomization of risks, and different ways of measuring risks may reshape the number and types of classes that emerge from the LCA models.

## Conclusion

We demonstrated that multiple disadvantages *within* the grandparental and parental generations were associated with comparably high probabilities of grandchild psychiatric disorders. This was also the case for grandchildren with combinations of grandparental socioeconomic disadvantages and parental psychosocial disadvantages. Importantly, improved socioeconomic and psychosocial circumstances *across* previous generations predicted comparably low probabilities of grandchild psychiatric disorders. Policies and interventions alleviating the burden of family disadvantages may have far-reaching implications for future generations’ mental health.

## Electronic Supplementary Material

Below is the link to the electronic supplementary material.

Supplementary Material 1

## Data Availability

Access to the data is restricted due to ethical regulations for the Stockholm Birth Cohort Multigenerational Study (SBC Multigen). If there is interest in the unpublished data from this research article, a request can be made to the main author, who will forward it to the steering committee of the SBC Multigen, and the committee will evaluate the request and decide on data to extract for specific purposes.
