# Supplementary material for: Psychiatric disorders following the clustering of family disadvantages in previous generations: a multigenerational cohort study
Source: Soc Psychiatry Psychiatr Epidemiol. 2025 Apr 29;61(1):119–31. doi: 10.1007/s00127-025-02918-z (PMC12855240; doi:10.1007/s00127-025-02918-z)
Supplement: Supplementary file 1 — Supplementary Material 1 [file 127_2025_2918_MOESM1_ESM.docx]

**Title: Psychiatric disorders following the clustering of family disadvantages in previous generations: A multigenerational cohort study**

**Journal name: Social Psychiatry and Psychiatric Epidemiology**

**Author names and affiliations:**

Baojing Li ^a^ (baojing.li@su.se); Can Liu ^a^ (can.liu@su.se); Ylva B. Almquist ^a^ (ylva.almquist@su.se); Lisa Berg ^a^ (lisa.berg@su.se)

^a^ Department of Public Health Sciences, Centre for Health Equity Studies (CHESS), Stockholm University, SE-106 91, Stockholm, Sweden

**Corresponding author:** Baojing Li (baojing.li@su.se), Department of Public Health Sciences, Centre for Health Equity Studies (CHESS), Stockholm University, SE-106 91, Stockholm, Sweden

**Online Resource 1. Fit statistics** **and information criterion for grandparental (G0) and parental latent class (G1) models with one to six latent classes.**

| **Number of classes** | | **AIC** | **BIC** | **SABIC** | **Entropy** | **VLMR-LRT**  **(P value)** | **Minimum class sizes**  **(proportions)** |
| --- | --- | --- | --- | --- | --- | --- | --- |
|  | 1 | 120574.651 | 120631.762 | 120609.516 | NA | NA | 25814  (100%) |
|  | 2 | 113804.966 | 113927.347 | 113879.677 | 0.696 | <.001 | 4512  (17.5%) |
| G0 | 3 | 112619.174 | 112806.823 | 112733.730 | 0.758 | <.001 | 1858  (7.2%) |
|  | 4 | 112510.930 | 112763.849 | 112665.332 | 0.785 | <.001 | 540  (2.1%) |
|  | 5 | 112467.718 | 112785.906 | 112661.965 | 0.817 | <.001 | 543  (2.1%) |
|  | 6 | 112452.279 | 112835.737 | 112686.372 | 0.667 | .13 | 528  (2.0%) |
|  | 1 | 127301.392 | 127358.503 | 127336.257 | NA | NA | 25814  (100%) |
|  | 2 | 123650.385 | 123772.765 | 123725.095 | 0.542 | <.001 | 4942  (19.1%) |
| G1 | 3 | 123234.415 | 123422.065 | 123348.971 | 0.668 | .01 | 1611  (6.2%) |
|  | 4 | 123079.393 | 123332.312 | 123233.795 | 0.566 | <.001 | 1202  (4.7%) |
|  | 5 | 123014.031 | 123332.219 | 123208.278 | 0.630 | <.001 | 466  (1.8%) |
|  | 6 | 122967.234 | 123350.691 | 123201.326 | 0.613 | .005 | 137  (1.7%) |

**Online Resource 2. Probabilities of transitioning from grandparental (G0) latent classes of family disadvantages to parental (G1) latent classes of family disadvantages. Results from latent transition analysis.**

| **G1**  **G0** | **H-SP** | **M-SP, H-TM** | **M-P, H-PA** | **M-SP, H-LI** | **N-SP** |
| --- | --- | --- | --- | --- | --- |
| **H-SP** | 0.125 *** | 0.034 * | 0.216 *** | 0.177 *** | 0.447 *** |
| **H-S** | 0.066 *** | 0.034 *** | 0.120 *** | 0.242 *** | 0.539 *** |
| **H-OC** | 0.038 *** | 0.025 *** | 0.141 *** | 0.178 *** | 0.619 *** |
| **M-SP** | 0.140 *** | 0.057 *** | 0.227 *** | 0.223 *** | 0.352 *** |
| **N-SP** | 0.022 *** | 0.009 *** | 0.090 *** | 0.150 *** | 0.729 *** |

*** *P* <.001, ** *P* <.01, * *P*<.05, estimates are statistically significantly different from 0.

For G0
H-SP: High-level socioeconomic and psychosocial disadvantages;
H-S: High-level socioeconomic disadvantages;
H-OC: High-level overcrowding;

M-SP: Medium-level socioeconomic and psychosocial disadvantages;
N-SP: No socioeconomic and psychosocial disadvantage;

For G1
H-SP: High-level socioeconomic and psychosocial disadvantages;
M-SP, H-TM: Medium-level socioeconomic and psychosocial disadvantages, with high-level teenage motherhood;
M-P, H-PA: Medium-level psychosocial disadvantages, with high-level single parenthood;
M-SP, H-LI: Medium-level socioeconomic and psychosocial disadvantages, with high-level low income;
N-SP: No socioeconomic and psychosocial disadvantage.

**Online Resource 3. Probabilities of psychiatric disorders among grandchildren (G2) by grandparental (G0) and parental (G1) latent classes of family disadvantages. Results from** **sensitivity analysis for the sample with equal length of follow up period for all G2s (e.g., up to age 30).**

| **Family classes** | | **Psychiatric disorders probability** | | |
| --- | --- | --- | --- | --- |
|  |  | **Total** | **Male** | **Female** |
|  |  | Probability (95% CI) | Probability (95% CI) | Probability (95% CI) |
|  | H-SP | 0.049 (0.026-0.072) | 0.049 (0.016-0.083) | 0.049 (0.016-0.081) |
|  | H-S | 0.085 (0.068-0.102) | 0.082 (0.058-0.105) | 0.089 (0.064-0.114) |
| G0 | H-OC | 0.062 (0.050-0.075) | 0.067 (0.050-0.085) | 0.057 (0.040-0.074) |
|  | M-SP | 0.087 (0.071-0.104) | 0.102 (0.078-0.127) | 0.071 (0.048-0.093) |
|  | N-SP | 0.046 (0.042- 0.050) | 0.046 (0.041-0.052) | 0.046 (0.041-0.052) |
|  | H-SP | 0.129 (0.094-0.164) | 0.175 (0.122-0.228) | 0.076 (0.033-0.119) |
|  | M-SP, H-TM | 0.049 (0.017-0.082) | 0.093 (0.035-0.151) | 0.007 (-0.023-0.036) |
| G1 | M-P, H-PA | 0.112 (0.090-0.134) | 0.120 (0.086-0.153) | 0.105 (0.075-0.134) |
|  | M-SP, H-LI | 0.062 (0.044-0.080) | 0.042 (0.019-0.065) | 0.087 (0.058-0.116) |
|  | N-SP | 0.039 (0.034-0.044) | 0.041 (0.033-0.049) | 0.037 (0.029-0.044) |

CI, confidence interval.

For G0
H-SP: High-level socioeconomic and psychosocial disadvantages;
H-S: High-level socioeconomic disadvantages;
H-OC: High-level overcrowding;

M-SP: Medium-level

socioeconomic and psychosocial disadvantages;
N-SP: No socioeconomic and psychosocial disadvantage;

For G1
H-SP: High-level socioeconomic and psychosocial disadvantages;
M-SP, H-TM: Medium-level socioeconomic and psychosocial disadvantages, with high-level teenage motherhood;
M-P, H-PA: Medium-level psychosocial disadvantages, with high-level single parenthood;
M-SP, H-LI: Medium-level socioeconomic and psychosocial disadvantages, with high-level low income;
N-SP: No socioeconomic and psychosocial disadvantage.

**Online Resource 4. Probabilities of psychiatric disorders among grandchildren (G2) by grandparental (G0) and parental (G1) latent classes of family disadvantages. Results from sensitivity analysis adjusting for the clustering/non-independence of observations within families.**

| **Family classes** | | **Psychiatric disorders probability** | | |
| --- | --- | --- | --- | --- |
|  |  | **Total** | **Male** | **Female** |
|  |  | Probability (95% CI) | Probability (95% CI) | Probability (95% CI) |
|  | H-SP | 0.078 (0.050-0.105) | 0.086 (0.047-0.125) | 0.070 (0.033-0.106) |
|  | H-S | 0.097 (0.080-0.114) | 0.087 (0.064-0.109) | 0.107 (0.082-0.132) |
| G0 | H-OC | 0.078 (0.065-0.091) | 0.081 (0.063-0.100) | 0.075 (0.057-0.093) |
|  | M-SP | 0.118 (0.099-0.138) | 0.126 (0.098-0.154) | 0.110 (0.084-0.136) |
|  | N-SP | 0.059 (0.055-0.063) | 0.055 (0.050-0.060) | 0.063 (0.057-0.069) |
|  | H-SP | 0.180 (0.141-0.219) | 0.210 (0.152-0.267) | 0.146 (0.096-0.195) |
|  | M-SP, H-TM | 0.127 (0.077-0.176) | 0.141 (0.063-0.219) | 0.113 (0.050-0.176) |
| G1 | M-P, H-PA | 0.139 (0.114-0.164) | 0.145 (0.108-0.182) | 0.132 (0.098-0.166) |
|  | M-SP, H-LI | 0.069 (0.052-0.087) | 0.062 (0.040-0.085) | 0.078 (0.052-0.104) |
|  | N-SP | 0.050 (0.045-0.056) | 0.046 (0.038-0.053) | 0.055 (0.047-0.063) |

CI, confidence interval.

For G0
H-SP: High-level socioeconomic and psychosocial disadvantages;
H-S: High-level socioeconomic disadvantages;
H-OC: High-level overcrowding;

M-SP: Medium-level socioeconomic and psychosocial disadvantages;
N-SP: No socioeconomic and psychosocial disadvantage;

For G1
H-SP: High-level socioeconomic and psychosocial disadvantages;
M-SP, H-TM: Medium-level socioeconomic and psychosocial disadvantages, with high-level teenage motherhood;
M-P, H-PA: Medium-level psychosocial disadvantages, with high-level single parenthood;
M-SP, H-LI: Medium-level socioeconomic and psychosocial disadvantages, with high-level low income;
N-SP: No socioeconomic and psychosocial disadvantage.

**Online Resource 5. Probabilities of psychiatric disorders among the total, male and female grandchildren (G2) for each unique transition pattern from grandparental (G0) latent classes of family disadvantages to parental (G1) latent classes of family disadvantages.**

| **Grandparental and parental family classes** | **Psychiatric disorders probability** | | |
| --- | --- | --- | --- |
|  | **Total** | **Male** | **Female** |
|  | Probability (95% CI) | Probability (95% CI) | Probability (95% CI) |
| **From G0: H-SP** | | | |
| **To G1: H-SP** | 0.279 (0.092-0.466) | 0.305 (0.058-0.552) | 0.239 (-0.043-0.521) |
| **G1: M-SP, H-TM** | 0.000 (0.000-0.000) | 0.000 (0.000-0.000) | 0.073 (-0.138-0.284) |
| **G1: M-P, H-PA** | 0.000 (0.000-0.000) | 0.000 (0.000-0.000) | 0.000 (0.000-0.000) |
| **G1: M-SP, H-LI** | 0.000 (0.000-0.000) | 0.051 (-0.178-0.279) | 0.000 (0.000-0.000) |
| **G1: N-SP** | 0.100 (0.035-0.165) | 0.093 (-0.019-0.204) | 0.106 (0.029-0.182) |
| **From G0: H-S** | | | |
| **To G1: H-SP** | 0.182 (0.039-0.324) | 0.140 (-0.056-0.336) | 0.219 (0.014-0.423) |
| **G1: M-SP, H-TM** | 0.140 (-0.025-0.305) | 0.171(-0.071-0.414) | 0.106 (-0.115-0.327) |
| **G1: M-P, H-PA** | 0.260 (0.119-0.402) | 0.306 (0.097-0.514) | 0.215 (0.022-0.409) |
| **G1: M-SP, H-LI** | 0.123 (0.050-0.196) | 0.074 (-0.015-0.163) | 0.173 (0.057-0.288) |
| **G1: N-SP** | 0.036 (0.000-0.071) | 0.035 (-0.011-0.081) | 0.036 (-0.019-0.091) |
| **From G0: H-OC** |  |  |  |
| **To G1: H-SP** | 0.123 (-0.057-0.302) | 0.225 (-0.217-0.666) | 0.078 (-0.088-0.244) |
| **G1: M-SP, H-TM** | 0.162 (-0.013-0.337) | 0.148 (-0.212-0.508) | 0.169 (-0.020-0.359) |
| **G1: M-P, H-PA** | 0.109 (0.028-0.191) | 0.142 (0.026-0.258) | 0.071 (-0.042-0.185) |
| **G1: M-SP, H-LI** | 0.066 (0.003-0.129) | 0.011 (-0.064-0.086) | 0.134 (0.026-0.241) |
| **G1: N-SP** | 0.069 (0.046-0.091) | 0.081 (0.050-0.113) | 0.055 (0.022-0.087) |
| **From G0: M-SP** |  |  |  |
| **To G1: H-SP** | 0.253 (0.155-0.351) | 0.303 (0.167-0.439) | 0.187 (0.050-0.323) |
| **G1: M-SP, H-TM** | 0.154 (0.031-0.278) | 0.228 (0.049-0.407) | 0.056 (-0.098-0.209) |
| **G1: M-P, H-PA** | 0.151 (0.066-0.236) | 0.169 (0.033-0.304) | 0.135 (0.030-0.240) |
| **G1: M-SP, H-LI** | 0.088 (0.005-0.171) | 0.084 (-0.039-0.206) | 0.093 (-0.018-0.204) |
| **G1: N-SP** | 0.055 (-0.002-0.112) | 0.032 (-0.048-0.112) | 0.081 (-0.001-0.164) |
| **From G0: N-SP** |  |  |  |
| **To G1: H-SP** | 0.093 (0.015-0.171) | 0.098 (-0.011-0.207) | 0.086 (-0.025-0.197) |
| **G1: M-SP, H-TM** | 0.095 (-0.010-0.201) | 0.044 (-0.110-0.197) | 0.148 (-0.001-0.297) |
| **G1: M-P, H-PA** | 0.137 (0.099-0.174) | 0.130 (0.077-0.183) | 0.144 (0.090-0.198) |
| **G1: M-SP, H-LI** | 0.059 (0.037-0.081) | 0.065 (0.036-0.095) | 0.051 (0.018-0.085) |
| **G1: N-SP** | 0.048 (0.042-0.054) | 0.042 (0.033-0.050) | 0.054 (0.045-0.062) |

CI, confidence interval.

For G0
H-SP: High-level socioeconomic and psychosocial disadvantages;
H-S: High-level socioeconomic disadvantages;
H-OC: High-level overcrowding;

M-SP: Medium-level socioeconomic and psychosocial disadvantages;
N-SP: No socioeconomic and psychosocial disadvantage;

For G1
H-SP: High-level socioeconomic and psychosocial disadvantages;
M-SP, H-TM: Medium-level socioeconomic and psychosocial disadvantages, with high-level teenage motherhood;
M-P, H-PA: Medium-level psychosocial disadvantages, with high-level single parenthood;
M-SP, H-LI: Medium-level socioeconomic and psychosocial disadvantages, with high-level low income;
N-SP: No socioeconomic and psychosocial disadvantage.

**Online Resource 6. Probabilities of psychiatric disorders among the total, male and female grandchildren (G2) for each unique transition pattern from grandparental (G0) latent classes of family disadvantages to parental (G1) latent classes of family disadvantages.** **Results from sensitivity analysis for the sample with equal length of follow up period for all G2s (e.g., up to age 30).**

| **Grandparental and parental family classes** | **Psychiatric disorders probability** | | |
| --- | --- | --- | --- |
|  | **Total** | **Male** | **Female** |
|  | Probability (95% CI) | Probability (95% CI) | Probability (95% CI) |
| **From G0: H-SP** | | | |
| **To G1: H-SP** | 0.137 (-0.023-0.296) | 0.238 (-0.005-0.481) | 0.000 (0.000-0.000) |
| **G1: M-SP, H-TM** | 0.000 (0.000-0.000) | 0.000 (0.000-0.000) | 0.000 (0.000-0.000) |
| **G1: M-P, H-PA** | 0.039 (-0.065-0.144) | 0.003 (-0.131-0.137) | 0.094 (-0.080-0.269) |
| **G1: M-SP, H-LI** | 0.000 (0.000-0.000) | 0.000 (0.000-0.000) | 0.063 (-0.167-0.294) |
| **G1: N-SP** | 0.060 (0.006-0.113) | 0.069 (-0.017-0.154) | 0.053 (-0.014-0.119) |
| **From G0: H-S** | | | |
| **To G1: H-SP** | 0.019 (-0.083-0.121) | 0.050 (-0.117-0.218) | 0.000 (0.000-0.000) |
| **G1: M-SP, H-TM** | 0.122 (-0.022-0.267) | 0.184 (-0.051-0.419) | 0.057 (-0.097-0.211) |
| **G1: M-P, H-PA** | 0.318 (0.172-0.464) | 0.326 (0.115-0.536) | 0.310 (0.107-0.513) |
| **G1: M-SP, H-LI** | 0.164 (0.077-0.252) | 0.112 (-0.005-0.228) | 0.210 (0.082-0.338) |
| **G1: N-SP** | 0.001 (-0.036-0.039) | 0.011 (-0.037-0.059) | 0.000 (0.000-0.000) |
| **From G0: H-OC** |  |  |  |
| **To G1: H-SP** | 0.002 (-0.152-0.156) | 0.036 (-0.351-0.424) | 0.000 (0.000-0.000) |
| **G1: M-SP, H-TM** | 0.044 (-0.070-0.159) | 0.062 (-0.221-0.345) | 0.035 (-0.050-0.120) |
| **G1: M-P, H-PA** | 0.129 (0.050-0.207) | 0.177 (0.060-0.293) | 0.074 (-0.028-0.175) |
| **G1: M-SP, H-LI** | 0.087 (0.015-0.158) | 0.004 (-0.072-0.079) | 0.196 (0.059-0.333) |
| **G1: N-SP** | 0.045 (0.023-0.067) | 0.059 (0.028-0.090) | 0.030 (-0.001-0.060) |
| **From G0: M-SP** |  |  |  |
| **To G1: H-SP** | 0.194 (0.104-0.285) | 0.250 (0.117-0.383) | 0.122 (0.010-0.234) |
| **G1: M-SP, H-TM** | 0.069 (-0.021-0.158) | 0.146 (-0.004-0.295) | 0.000 (0.000-0.000) |
| **G1: M-P, H-PA** | 0.088 (0.016-0.159) | 0.069 (-0.039-0.178) | 0.106 (0.012-0.200) |
| **G1: M-SP, H-LI** | 0.072 (-0.012-0.157) | 0.070 (-0.056-0.195) | 0.075 (-0.035-0.186) |
| **G1: N-SP** | 0.053 (-0.002-0.108) | 0.066 (-0.015-0.147) | 0.040 (-0.033-0.113) |
| **From G0: N-SP** |  |  |  |
| **To G1: H-SP** | 0.118 (0.035-0.201) | 0.124 (0.007-0.240) | 0.110 (-0.004-0.225) |
| **G1: M-SP, H-TM** | 0.020 (-0.049-0.089) | 0.026 (-0.091-0.142) | 0.014 (-0.055-0.083) |
| **G1: M-P, H-PA** | 0.096 (0.061-0.131) | 0.110 (0.057-0.164) | 0.082 (0.037-0.128) |
| **G1: M-SP, H-LI** | 0.040 (0.016-0.064) | 0.037 (0.007-0.068) | 0.044 (0.005-0.083) |
| **G1: N-SP** | 0.039 (0.033-0.045) | 0.038 (0.029-0.047) | 0.041 (0.032-0.049) |

CI, confidence interval.

For G0
H-SP: High-level socioeconomic and psychosocial disadvantages;
H-S: High-level socioeconomic disadvantages;
H-OC: High-level overcrowding;

M-SP: Medium-level socioeconomic and psychosocial disadvantages;
N-SP: No socioeconomic and psychosocial disadvantage;

For G1
H-SP: High-level socioeconomic and psychosocial disadvantages;
M-SP, H-TM: Medium-level socioeconomic and psychosocial disadvantages, with high-level teenage motherhood;
M-P, H-PA: Medium-level psychosocial disadvantages, with high-level single parenthood;
M-SP, H-LI: Medium-level socioeconomic and psychosocial disadvantages, with high-level low income;
N-SP: No socioeconomic and psychosocial disadvantage.

**Online Resource 7. Probabilities of psychiatric disorders among the total, male and female grandchildren (G2) for each unique transition pattern from grandparental (G0) latent classes of family disadvantages to parental (G1) latent classes of family disadvantages.** **Results from sensitivity analysis adjusting for the clustering/non-independence of observations within families.**

| **Grandparental and parental family classes** | **Psychiatric disorders probability** | | |
| --- | --- | --- | --- |
|  | **Total** | **Male** | **Female** |
|  | Probability (95% CI) | Probability (95% CI) | Probability (95% CI) |
| **From G0: H-SP** | | | |
| **To G1: H-SP** | 0.279 (0.102-0.456) | 0.305 (0.052-0.558) | 0.239 (-0.022-0.500) |
| **G1: M-SP, H-TM** | 0.000 (0.000-0.000) | 0.000 (0.000-0.000) | 0.073 (-0.138-0.284) |
| **G1: M-P, H-PA** | 0.000 (0.000-0.000) | 0.000 (0.000-0.000) | 0.000 (0.000-0.000) |
| **G1: M-SP, H-LI** | 0.000 (0.000-0.000) | 0.051 (-0.180-0.282) | 0.000 (0.000-0.000) |
| **G1: N-SP** | 0.100 (0.034-0.166) | 0.092 (-0.018-0.202) | 0.106 (0.025-0.186) |
| **From G0: H-S** | | | |
| **To G1: H-SP** | 0.182 (0.054-0.310) | 0.140 (-0.043-0.323) | 0.219 (0.029-0.409) |
| **G1: M-SP, H-TM** | 0.140 (-0.094-0.374) | 0.171(-0.221-0.563) | 0.106 (-0.114-0.326) |
| **G1: M-P, H-PA** | 0.260 (0.112-0.408) | 0.305 (0.108-0.503) | 0.215 (0.021-0.409) |
| **G1: M-SP, H-LI** | 0.123 (0.051-0.196) | 0.074 (-0.015-0.162) | 0.173 (0.054-0.292) |
| **G1: N-SP** | 0.035 (-0.001-0.072) | 0.035 (-0.011-0.081) | 0.036 (-0.019-0.091) |
| **From G0: H-OC** |  |  |  |
| **To G1: H-SP** | 0.122 (-0.066-0.311) | 0.224 (-0.228-0.677) | 0.078 (-0.085-0.241) |
| **G1: M-SP, H-TM** | 0.162 (-0.010-0.333) | 0.148 (-0.213-0.508) | 0.169 (-0.023-0.362) |
| **G1: M-P, H-PA** | 0.109 (0.024-0.194) | 0.142 (0.016-0.267) | 0.071 (-0.041-0.183) |
| **G1: M-SP, H-LI** | 0.066 (0.003-0.130) | 0.011 (-0.065-0.086) | 0.134 (0.026-0.241) |
| **G1: N-SP** | 0.069 (0.046-0.092) | 0.081 (0.049-0.114) | 0.055 (0.022-0.087) |
| **From G0: M-SP** |  |  |  |
| **To G1: H-SP** | 0.253 (0.141-0.365) | 0.303 (0.141-0.465) | 0.187 (0.055-0.319) |
| **G1: M-SP, H-TM** | 0.154 (0.032-0.277) | 0.228 (0.053-0.403) | 0.056 (-0.098-0.210) |
| **G1: M-P, H-PA** | 0.151 (0.059-0.244) | 0.169 (0.014-0.323) | 0.135 (0.026-0.245) |
| **G1: M-SP, H-LI** | 0.088 (0.001-0.176) | 0.084 (-0.044-0.212) | 0.093 (-0.018-0.204) |
| **G1: N-SP** | 0.055 (-0.004-0.114) | 0.032 (-0.053-0.116) | 0.081 (-0.002-0.164) |
| **From G0: N-SP** |  |  |  |
| **To G1: H-SP** | 0.093 (0.010-0.176) | 0.098 (-0.017-0.213) | 0.086 (-0.025-0.196) |
| **G1: M-SP, H-TM** | 0.095 (-0.010-0.200) | 0.044 (-0.111-0.198) | 0.147 (-0.001-0.296) |
| **G1: M-P, H-PA** | 0.137 (0.098-0.176) | 0.130 (0.075-0.185) | 0.144 (0.090-0.198) |
| **G1: M-SP, H-LI** | 0.059 (0.036-0.083) | 0.065 (0.035-0.095) | 0.051 (0.017-0.085) |
| **G1: N-SP** | 0.048 (0.041-0.054) | 0.042 (0.033-0.050) | 0.054 (0.045-0.063) |

CI, confidence interval.

For G0
H-SP: High-level socioeconomic and psychosocial disadvantages;
H-S: High-level socioeconomic disadvantages;
H-OC: High-level overcrowding;

M-SP: Medium-level socioeconomic and psychosocial disadvantages;
N-SP: No socioeconomic and psychosocial disadvantage;

For G1
H-SP: High-level socioeconomic and psychosocial disadvantages;
M-SP, H-TM: Medium-level socioeconomic and psychosocial disadvantages, with high-level teenage motherhood;
M-P, H-PA: Medium-level psychosocial disadvantages, with high-level single parenthood;
M-SP, H-LI: Medium-level socioeconomic and psychosocial disadvantages, with high-level low income;
N-SP: No socioeconomic and psychosocial disadvantage.
